# Introduction: The social life of time

**DOI:** 10.1177/0961463X20921674

**Published:** 2020-05-07

**Authors:** Michelle Bastian, Lisa Baraitser, Michael J Flexer, Andrew R Hom, Laura Salisbury

**Affiliations:** University of Edinburgh, Edinburgh, UK; Birkbeck, University of London, London, UK; University of Exeter, Exeter, UK; University of Edinburgh, Edinburgh, UK; University of Exeter, Exeter, UK

In recent social science literatures, a focus on the “social life” of something suggests an interest in foregrounding the active capacities of what has traditionally been thought of as inert. Approaches drawing on Arjun Appadurai’s framework (1988) look to follow the movement of things such as an object, a device, or a method, through its everyday manifestations in order to understand how it takes part in producing social life. This suggests that in challenging accounts of time as an inert background to social life, we might want to focus on the agency of particular manifestations of time, and also attempt to situate them by tracing them through particular contexts and everyday activities.

A paper on “The double social life of methods” by John Law et al. (2011) provides us with further avenues for thinking through, about, alongside, and from within, the social life of time. Law et al. argue that understanding research methods as having social lives requires two moves. The first, which they take to be relatively unremarkable, is to claim that “methods are social because they are constituted by the social world of which they are a part” (Law et al., 2011: 4). They claim that methods are not neutral but have a purpose and they have advocates. They “embody the concerns of advocates and subsist in particular contexts or environments” (Law et al., 2011: 7); that is, methods are *of* the social. While this first move may be unremarkable when it comes to methods, if we think about making similar claims about time then, outside of the community drawn together by this journal, we can often find ourselves working very much against the grain. Accounts of time as having a purpose? As having particular advocates? As requiring particular social and environmental ecologies in order to manifest? How do questions like these fit with taken-for-granted concepts of time as objective and universal?

The second move made by Law et al., which is taken to be more controversial, is that methods also “constitute and organise” the social (2011: 8). They are not just produced by the social; rather, research methods also in turn *produce* the social. Here, we would appear to be on more familiar territory when it comes to time. That time organizes the social – even makes the social – is a less remarkable claim to make, particularly if the focus is on time-keeping, or shared histories and futures. However, for a social life of time, we need to understand both of the moves suggested by Law et al. as working together: that is, in order to understand the social life of time we need to hold together both the ways that time *organizes* the social and that time is *of* the social. If we do not understand time as *of* the social, then the politics of time, the politics of time’s role in organizing the social worlds that also constitute it, remains hidden.

A key interest of this special issue is in social and cultural processes of discrimination and transformation. This double focus, supported by an enquiry into time’s “social life”, enables a more critical look at the way time produces and performs some realities while shutting down others, precisely through the way it organizes and constitutes social life. This is clear in contributor Charles W Mills’ earlier essay (2014) on “White Time,” for example, where he discusses the “white temporal imaginary” and its production of exclusionary temporal ghettos. Times do have purposes and advocates, both explicit and implicit. They also have critics.

Finally, a third move in thinking through time’s social life – in addition to the two suggested by Law et al. – is to turn again to how we define time. While as editors of this special issue, we come to questions of time and temporality from a wide variety of human sciences, we coalesce around the idea that it is important to challenge the idea that time is, at its most fundamental, about a natural flow, about the pace of this flow or about the various ways that past, present and future might mesh together within a temporal flow. Time as movement, change, rupture, or speed is no doubt important, but another focus that this special issue takes up is the ways these aspects of time themselves arise from particular relational configurations, interrelations and dependencies. As Bruno Latour argues in his essay “Trains of Thought” (2005), time flows in particular ways due to specific alignments of humans and nonhumans in relation, but the flow of time is not itself a primary phenomenon, rather the relations are. Importantly, Latour notes that these configurations are not equally beneficial to all. So what might we make of a definition of *time as uneven and unequal relationality*, rather than time as flow?

With this definition in hand, the work on the social life of time in this issue asks questions such as: What form of relationality is a particular manifestation of time enacting? Who is included and who is excluded? Who appears, who disappears? Who has agency and who doesn’t? What entities are aligned, and in what ways, in order for this experience of time to arise? Why one particular uneven configuration and not others? Who benefits and who suffers? And who gets to decide?

This special issue emerges from an international interdisciplinary conference titled *The Social Life of Time: power, discrimination and transformation* that took place at the University of Edinburgh in June 2018. The conference was a collaboration between *Temporal Belongings*, an AHRC-funded research network that explores the relationship between time and communities, and *Waiting Times*, a Wellcome Trust-funded research project that investigates the relation between time and care, and in particular healthcare. With keynotes by Charles W Mills, jackie sumell, Judy Wajcman and Paul Huebener, the conference opened up the questions above, challenging dominant approaches to time across a range of disciplines. The articles collected here represent a vibrant sample of papers presented at this event. Organized into three sections – politics, health and the life course and waiting and remaining – this special issue showcases a “critical time studies” (Huebener, 2015) which takes issues of power, discrimination and transformation to be absolutely central to the question of “what is time?”

Each section, which we introduce below, hangs together around a central social domain or space. However, readers will also find many productive themes cutting across the sections. For instance, authors raise questions around the emancipatory possibilities of time (Mills, Kennedy, Flexer), thematizing medicalized and pathological times, which are then explored in greater detail in terms of time made as scientific measurement (Marathe, Clark). Measuring and accounting for human value leads us into questions of time’s structuring of particular social, material, and experiential inequalities (Fraenkel, Wanka, Harris and Coleman, Ringel). More particular forms of temporal punishment are raised in terms of excluded communities, including migrants, temporary workers and the dispossessed (Drangsland, Harper and Zubida, Sa’di-Ibraheem). A further thematic highlights the interplay between time and power (McIntosh, Porschy).

## Politics

In our first section, time is intensely and perhaps intrinsically political – it suborns power, and vice versa. Time regimes make and unmake subjects, elevating certain interests and subordinating others. Temporal claims offer political advantage to elite actors and authorities. **Charles W Mills**’ keynote presentation connects chronopolitics to Zerubavel’s “time maps” in order to further develop his account of racial time through an inquiry into problematic periodizations, for example “white modernity.” His vigorous critique of that dominant temporal trope concludes with a rousing call to recalibrate and destabilize the “Euro-chronometer” in hopes of discovering a less violent and oppressive politics of time.

Political power also operates through imposed waiting times. **Kari Anne Drangsland** uses ethnographic methods to examine the embedded temporal politics of an offer of possible legalization that the Hamburg government gave to a group of 350 illegalized West-African migrants in Germany in 2013. She shows how waiting in time, and the seemingly just-around-the-corner prospect of political redemption in the form of legal status, combined to govern migrants in a “lived timespace.” She concludes that temporal governance works hand in glove with territorial dominance to periodize and fix vulnerable subjects.

**Yara Sa’di-Ibraheem** addresses the changes of temporalities within an even more prolonged displacement process, which has been taking place in the Palestinian city of Jaffa while under Israeli rule. She tracks how Israeli authorities imposed various temporal perspectives on newly emptied but historically Palestinian neighbourhoods, including claims about *terra sine tempore* and “ahistorical” stories that worked to freeze and eventually expel residents. Former residents then in turn experienced political time as indefinite waiting, as perpetual uncertainty, followed by the need to rush – all of which left them with a sense of being “out of time” in multiple ways.

Powerful actors mobilize time in a wide variety of ways beyond deferral and waiting. **Chris McIntosh** uses an analytical focus on “the present” as the locus of experience to unpack the timing moves that help Donald Trump’s Presidency to produce an indefinite sense of “now,” regimes of temporal othering, and a seemingly unmanageable pace of political activity. These elements encourage acquiescence even from staunch critics and augur, in McIntosh’s argument, an increasingly unpredictable and violent future for international politics. Finally, **Jürgen Portschy** provides an outline of time’s relationship to political power through close readings of Michel Foucault’s work, which is sometimes considered more interested in space and territory than time and history. Portschy finds Foucault deeply invested in the analysis of particular historically dominant – but also fundamentally contested – social time regimes, which help account for both ruptures and continuities in lived experience. While highlighting the temporal Foucault, Portschy also offers us a distinctly Foucauldian lexicon of time. Taken together, these articles leave little doubt that where we find politics, in all its varied forms, we are likely to find time and temporality doing important conceptual and practical work in the service of governance and contestation.

## Health and the life course

Questions of the relation between time and care, and in particular healthcare, emerge in our second section, and particularly in its first paper by **Megh Marathe**. In a discussion of clinical processes involved in diagnosing epileptic seizures, they show how internalized clock time norms inform clinicians in their daily practice of distinguishing between different representations of brain waves. In doing so, they argue, a set of aesthetics are assigned to brain waves in which clock-time norms are beautiful and hard-to-classify patterns are ugly. In turn, this aestheticization of time has material effects that cover over the labour and suffering of living with regular and unpredictable seizures. By showing how time shapes care, Marathe calls for a situated and collaborative process between clinicians and patients that can capture lived experiences in clinical care that otherwise remain occluded.

**Michael Flexer** asks how two late 20th-century texts use literary form to explore a link between ideas of mental time travel and lived experiences of psychosis. Exploring the uncertainty within the texts’ use of deictic terms (like “I”, “here”, or “now”) and their effects on the orientation of the protagonists’ subjective experience, Flexer suggests how these literary texts offer insights into lived experiences of “psychotic” temporal disruption. By aligning these insights with clinical observations, he articulates the possibility of a form of care linked to understanding these deictic arrangements not as essentially disturbing, but as having a particular, legible temporal structure.

Moving from mental time travel to mental speed, **Justin Tyler Clark** explores the rise of “quick thinking” as a sign of intelligence. Identifying a cross-fertilization between stenography, telegraphy and psychology via the notion of “words per minute,” he shows how the adoption of timed testing redefined intelligence. In this case of the social life of methods, where the search for efficient tests led to reconstituted social understandings of the mind, also reconstitutes social understandings of time.

In the final paper in this section, the question of how the time of a life course gets framed and rendered normative is explored by **Anna Wanka**’s analysis of the use of chronological age to structure life transitions. Using empirical research, she shows how, in retirement, an understanding of the limits of time can reframe and “queer” normative temporal expectations.

## Waiting and remaining

In the final section of this special issue, we focus on the social life of that which remains and those who are made to wait. **Tanya Ann Kennedy** interrogates the persistence of “white time” in cultural and critical imaginaries, critiquing the models of crisis, backlash and progress that have been central to white feminist narratives. By focusing on the Combahee River Collective and the National Women’s Conference from 1977, she parses out a commitment to another kind of future – of “what could have been” and what yet may be – from black feminist thought.

**Robin A Harper and Hani Zubida** use qualitative research with temporary labour migrants in Israel to understand what it means to enter and use “migration time.” Noting generational differences between migrants and their children and cultural distinctions across migrant groups, they track the affective shifts and complex identifications that emerge within and from multiple non-linear timescapes.

Moving to a focus on the material infrastructures of the city, and resonating with Sa’di-Ibraheem’s interest in the built environment, **Felix Ringel** analyses the clashing times of decay, maintenance and gentrification in the Goetheviertel district of Germany’s Bremerhaven. Emphasizing the intertwining of the social *and* material lives of time, Ringel draws attention to the temporal agency of the deteriorating houses of the district and how they coproduce, thwart, and offer different potential futures for current and prospective residents of the area.

In his contribution to this special issue, **Peter Fraenkel** critically questions the notion of waiting by focusing on homeless families in New York. Far from simply passively waiting for the provision of state support, as they have been caricatured as doing, Fraenkel identifies multiple temporal challenges that need to be negotiated in order to try to maintain family bonds. Highlighting contradictions between the multiple institutions the research participants must engage with, this paper seeks ultimately to promote more complex understandings of the time binds faced by service users amongst those who work to support them.

We conclude this special issue with an article that takes us back to the question of “the social life of methods” with which we began this introduction and asks how this might apply to the methods we use to study time. Here, **Ella Harris and Rebecca Coleman** reflect on their use of quite different methodological approaches, using digital methods and walking methods respectively. Their interest is in drawing out how these methods can reveal the particular infrastructures that support some social rhythms and temporal logics over others. In doing so, they highlight how these methods enable us to approach the question of how time takes part in producing social life.
